# Maternal Protein Malnutrition: Current and Future Perspectives of Spirulina Supplementation in Neuroprotection

**DOI:** 10.3389/fnins.2018.00966

**Published:** 2018-12-18

**Authors:** Shrstha Sinha, Nisha Patro, Ishan K. Patro

**Affiliations:** ^1^School of Studies in Neuroscience, Jiwaji University, Gwalior, India; ^2^School of Studies in Zoology, Jiwaji University, Gwalior, India

**Keywords:** protein malnutrition, fetal programming, oxidative stress, inflammation, nutritional supplementation, Spirulina, neuroprotectant

## Abstract

Malnutrition has been widely recognized as a grave burden restricting the progress of underdeveloped and developing countries. Maternal, neonatal and postnatal nutritional immunity provides an effective approach to decrease the risk of malnutrition associated stress in adulthood. Particularly, maternal nutritional status is a critical contributor for determining the long-term health aspects of an offspring. Maternal malnutrition leads to increased risk of life, poor immune system, delayed motor development and cognitive dysfunction in the children. An effective immunomodulatory intervention using nutraceutical could be used to enhance immunity against infections. The immune system in early life possesses enormous dynamic capacity to manage both genetic and environment driven processes and can adapt to rapidly changing environmental exposures. These immunomodulatory stimuli or potent nutraceutical strategy can make use of early life plasticity to target pathways of immune ontogeny, which in turn could increase the immunity against infectious diseases arising from malnutrition. This review provides appreciable human and animal data showing enduring effects of protein deprivation on CNS development, oxidative stress and inflammation and associated behavioral and cognitive impairments. Relevant studies on nutritional supplementation and rehabilitation using Spirulina as a potent protein source and neuroprotectant against protein malnutrition (PMN) induced deleterious changes have also been discussed. However, there are many futuristic issues that need to be resolved for proper modulation of these therapeutic interventions to prevent malnutrition.

## Introduction

Rapid industrialization and commercialization have changed our living standard and dietary habits. A proper nutrition provides a flexible infrastructure for the brain development which forms the basis of human growth and economic progress. The hunger level is measured globally as Global Hunger Index (GHI) by Welthungerhilfe and concern worldwide to evaluate the progression in hunger (Von Grebmer et al., 2018). 12.3% of world’s population is undernourished with 27.9% stunted and 9.3% wasted children as reported by GHI, 2018. Every country in the world is facing a grave burden of malnutrition where South Asia and Africa is striving hard with under serious hunger category with GHI of 30.5 and 29.4, respectively. The mortality rate of children aged under five, category is 4.2%. However, GHI score of almost all countries from 2000 to 2018 decreased substantially except Central African Republic which shows no advancement. India ranks 103 out of 119 countries assessed with a GHI score of 31.1 (serious category). Still today, 20–50% of the patients admitted in hospital are found to be malnourished (Kirkland et al., 2013) with poor recovery rate from illness (Marshall et al., 2014). In simple words, malnutrition is a condition of imbalanced or inadequate nutrient intake by a person. It includes undernutrition (wasting, stunting, and underweight), over-nutrition (overweight and obesity) and micronutrient related malnutrition (micronutrient deficiency or excess). Malnutrition is a serious health problem associated with the increased susceptibility to mortality and morbidity. It is now widely accepted as silent executioner. In this review, we use the term ‘malnutrition’ to refer simply to a deficiency of nutrition particularly protein. Developmental Origins of Health and Disease (DOHAD) hypothesis suggest the link between early life developmental patterns and late onset of diseases (Barker et al., 2002; Shih et al., 2004). Many epidemiological and experimental reports suggest that nutritional status during fetal development plays an important role in maintaining energy metabolism at later life (Silveira et al., 2007; McMillen et al., 2008). The nutritional status of the mother strongly affects the brain development and cognitive abilities in the offspring. All the members in a community are affected by maternal nutritional patterns, but children and infant are at the highest risk as they need more nutrition for growth and development. Similarly, pregnant and lactating women are at risk and the babies born will be more prone to any disease at later life. Maternal and infant malnutrition is found to be associated with behavioral and cognitive impairment throughout the childhood and adulthood that makes them more vulnerable to neuropsychiatric disorders (Naik et al., 2015). Several studies demonstrated that maternal malnutrition alters the fetal genome and increases the risk of neuropsychiatric disorders including depression, schizophrenia, aggression, hyperactivity and anti-social behavior (Duran et al., 2005; Galler et al., 2005). In addition, it also reduces the thickness of the visual cortex, parietal neocortex, dentate gyrus, CA3 and cerebellum (Noback and Eisenman, 1981; Díaz-Cintra et al., 1990; Ranade et al., 2012). The compromised physical development, impaired spatial learning and memory, compromised astrogenesis and oligodendrogenesis leading to hypo-myelination have all been reported in an intra-generational protein restriction rat model (Naik et al., 2015, 2017; Patro et al., 2018). Protein malnutrition (PMN) is also found to be associated with free radical overproduction, decreased antioxidant defense system (Feoli et al., 2006; Khare et al., 2014) and immune impairments that initiate a cascade of inflammatory reaction (Welsh et al., 1998; Yamada et al., 2016).

Despite the high prevalence of the serious consequences of maternal PMN, a very little information dealing with potent nutritional rehabilitation strategies against malnutrition is available. There is an urgent need of food or a food supplement which imparts both nutritional and medicinal benefits to the society. Such types of dietary supplements are called as nutraceuticals or functional foods. Nowadays, microalgae are gaining popularity as a dietary supplement because of low cost, high nutritional value and enormous health benefits. Spirulina contains significant amount of proteins, vitamins, beta-carotene, minerals, polysaccharides, glycolipids and sulfolipids (Campanella et al., 1999; Blinkova et al., 2001; Watanabe et al., 2002; Colla et al., 2004). Various bioactive peptides are now derived from Spirulina which can be used as efficient nutraceutical ingredients in novel food designing (Ovando et al., 2018). A large number of studies evidence the neuroprotective role of Spirulina in variety of diseases like ischemic brain damage, Parkinson’s disease, LPS induced inflammation and rheumatoid arthritis (Bhat and Madyastha, 2001; Stromberg et al., 2005; Patro et al., 2011). Thus, dietary complementation with Spirulina could be a cost-effective way to enhance the balanced food security in a natural way. This review is focused on the effects of malnutrition on fetal programming, CNS development, oxidative stress, inflammation and various nutritional supplementation and rehabilitation strategies with special attention on neuroprotective role of Spirulina against PMN induced changes.

## Maternal Nutrition and Fetal Programming

The development of mammalian central nervous system is a complex process that begins *in utero* and continues throughout the adolescence and adulthood. It is tightly regulated by both genetic and environmental factors. The prenatal and postnatal environment affects the brain development, maturation and function both positively and negatively depending on the type of environmental stimuli (Kolb and Whishaw, 1998; Lenroot and Giedd, 2008). Several studies were conducted using different animal models, protocols, sources and types of enrichment to analyze how prenatal environmental enrichment (EE) affects the development of fetus (Kiyono et al., 1985; Mychasiuk et al., 2012; Rosenfeld and Weller, 2012). In most of the studies, EE is reported to exhibit beneficial effects on neuronal growth and maturation, neurocognitive abilities, cortical thickness, dendritic arborization, synaptic integrity, vascular inflammation, and neurogenesis (Nilsson et al., 1999; Olson et al., 2006; Reynolds et al., 2010; Simpson and Kelly, 2011; Mesa-Gresa et al., 2013; Caporali et al., 2014). Such favorable effects appears to be a common denominator for the remedial efficacy of an EE against experimental pathologies including trauma, cerebral ischemia, astroglial degeneration, and glioma growth (Passineau et al., 2001; Dahlqvist et al., 2004; Anastasía et al., 2009; Yuan et al., 2009; Cao et al., 2010; Jain et al., 2013; Rodriguez et al., 2013; Garofalo et al., 2015). Both the embryological and the postnatal development of the offsprings are governed by *in utero* environment (Barbazanges et al., 1996; O’donnell et al., 2009). An enriched *in utero* environment probably helps the fetus to survive in a specific environment as newborns are more prone to a variety of infections. Neonatal infection is a predominant cause of childhood mortality and morbidity causing 40% of mortality in under five age group (Liu et al., 2012; Bhutta and Black, 2013; Blencowe et al., 2013; Lawn et al., 2014).

Any adverse environmental condition imposed during the most vulnerable period of development (gestational and lactational period) affects the epigenetic status and also alter the covalent modifications of the DNA and histones of embryos, fetuses, and neonates (Reynolds and Caton, 2012; Wu et al., 2012). These changes may influence the complete life cycle of an offspring and may be transmitted from one generation to another and thus could result in the origin of *‘fetal programming*’ or ‘*neonatal programming*’ concept (Barker and Clark, 1997; Ji et al., 2016). Imbalanced maternal nutrition often leads to the pathophysiological condition termed as intrauterine growth restriction (IUGR). It exerts detrimental effects on preweaning survival and developmental rate, growth rate, food intake, organ structure and function (Wu et al., 2006; Rekiel et al., 2014) and onset of diseases at adulthood and senility, including type-2 diabetes, hypertension, hyperglycemia, and cardiovascular disease (Alberti et al., 2005; Longo et al., 2013; Langley-Evans, 2015). It also affects the transfer of nutrients and oxygen from pregnant mother to fetus, vascular growth and functional capacity of placenta (Wu et al., 2004; Leddy et al., 2008). Maternal stress or infection during the prenatal or perinatal period also alters the physiological and behavioral profile of the stress response progression in offspring. One important pathway for the transmission of prenatal stress is through the secretion of corticosterone from mother to fetus via placenta, a proximal symbol of the outer environment. Prenatal inhibition of maternal corticosterone secretion exerts no difference between progeny of stressed and non-stressed mothers, thus signifying a possible mechanism of maternal stress transmission via corticosterone (Barbazanges et al., 1996; Cicchetti, 2016). The placenta plays a key role in maternal nutrient transport to the fetus, thus placental abnormalities may inhibit the nutrient support to fetus. The nutrient supply depends on the size of placenta, morphology, blood flow and nutrient transporters (Fowden et al., 2006; Higgins et al., 2011). Thus, proper modulation of placental function either through gene expression or supplementation (nutrients or hormones) is necessary to fulfill the energy demands of growing fetus (Belkacemi et al., 2010). Both undernutrition and overnutrition contributes to the metabolic syndrome and chronic life-threatening diseases in adults (Boney et al., 2005; Leddy et al., 2008; Rkhzay-Jaf et al., 2012). To elucidate this, a number of studies have been conducted across the world using rat as a model organism to mimic the conditions of protein calorie malnutrition, total calorie undernutrition, maternal protein restriction and anemia (Ozanne and Hales, 2002; Armitage et al., 2004). Out of all these models, maternally protein deprived rat model is the most extensively studied model which involves the feeding of a low protein diet (5–8% protein) to pregnant dams in comparison to dams fed with a control diet (20% protein). The F1 progeny born from protein restricted mothers presented low birth weight and were more prone to cardiovascular diseases and psychiatric disorders (Fernandez-Twinn et al., 2003). Moreover, maternal over-nutrition before and during gestation also resulted in abnormal weight gain during pregnancy as compared to the normally nourished mothers which further increases with each successive pregnancy (Castro and Avina, 2002). Such anomalies further contribute to the progression of gestational diabetes along with long lasting negative health consequences for the infant (Ostlund et al., 2004). The excess protein supplementations to pregnant mothers also result in preterm delivery and enhanced perinatal mortality (Say et al., 2003). It is also observed that high fat diet during pregnancy decreases the mitochondrial copy number in kidney, thus changing the glucose homeostasis leading to a condition in parallel with mammalian metabolic syndrome counting endothelial dysfunction, hypertension, altered serum lipid status and adiposity (Armitage et al., 2005). Similarly, maternal glycemia also result in increased body weight and chances of diabetes in offspring (Franks et al., 2006).

## Protein Malnutrition and CNS Development

The proper placental development, fetus growth and all other changes occurring in mother’s body during pregnancy necessitate amino acids for protein formation. Both mother and fetus are actually competing for this ‘scarce resource.’ Major proportions of the amino acids in the fetus are transported from the maternal circulation through active transport (Regnault et al., 2005a,b). Both placenta and fetal tissues are the disposal sites of amino acids present in the fetus, which is further involved in fetal amino acid metabolism (Brown et al., 2011). Thus, because of great involvement of amino acid in CNS functioning (amino acid as precursors of neurotransmitter, structural proteins, enzymes, peptide hormones), studies on the effect of PMN in developing CNS is gaining acclamation. Generally, casein content is modified in the diet to design protein restricted animal model. The protein deprived diet consists of 5–9% protein, while 16–25% protein is present in the normal diet.

Brain development is tightly regulated by both environmental and genetic factors. The period of rapid brain growth is considered as most critical period of development as brain is more vulnerable to any insult during this period. Any insult incurred during this period of brain development may result in long lasting negative effects on brain function, behavior and cognition. Nutrition is one of the most important epigenetic regulators that can affect brain function and behavior. Nutritional insults during pregnancy changes the epigenetic of the fetal genome and may leave a permanent devastating effect. The severity of the effects depends on the developmental timeline and magnitude of insult, i.e., earlier the insult, the more permanent the effects are. Vast literature indicate that malnutrition influences the cell count, synapse formation, dendritic arborization, cellular differentiation and proliferation, cell migration, growth factor synthesis and myelination and all these changes in turn result in impaired motor and cognitive functions (Georgieff, 2007; Prado and Dewey, 2014). Other studies also reported a reduction in dendritic basal number and processes, spine density, thickness of dendrites, somal size and neuronal loss following malnutrition (Salas and Nieto, 1974; Garcia-Ruiz et al., 1993; Lister et al., 2005). Such changes in malnourished animals may lead to permanent disturbances in dendritic arborization, architecture and synaptic efficiency (West and Kemper, 1976; Desmond and Levy, 1988). *In utero* protein deprivation also leads to reduced body and brain weight (Dickerson et al., 1967; Smart et al., 1973; Peeling and Smart, 1994). In nutshell, the nutritional inadequacies affect the neuroanatomy, neurochemistry, neurophysiology and neuropathology of the nervous system. A balanced nutrient supply which includes protein, iron, zinc, folate, choline, vitamin A and polyunsaturated fatty acids, governs brain function, behavior and cognitive development (Rao and Georgieff, 2001). Rats born to protein deprived mothers have shown increased arterial blood pressure and high cardiovascular sympathetic tone (Ozaki et al., 2001; Barros et al., 2015). It is observed that periconceptional and prenatal nutritional insult affects the postnatal brain maturational events (Morgane et al., 2002). Early life nutritional insult is also reported to be associated with enhanced risk for schizophrenia development (Brown and Susser, 2008; Bale et al., 2010). Hippocampus is adversely affected by early malnutrition. A significant reduction in the size of cells of the dentate gyrus, reduction in the degree of dendritic branching, and reduction in the number of granule cells is evident in hippocampus (Levitsky and Strupp, 1995).

Salas and Nieto (1974) reported that PMN also affects the size of cerebellum and dendrites of cortical pyramidal cells. Cell generation time is also increased in undernourished conditions (Deo et al., 1978). In cerebellar cortex, granule cells and basket cells per Purkinje neuron were reduced in number along with hypoplasia of glial cell following PMN (Clos et al., 1977). Severe lactational undernutrition is also reported to decrease the number of cells both in external germinal layer and internal granular layer (Barnes and Altman, 1973).

More recently, Gould et al. (2018) have hypothesized that maternal PMN before implantation can cause the adverse developmental programming leading to behavioral deficits and short-term memory loss. This was correlated with reduced neural stem cell and progenitor cell numbers through suppressed proliferation, defective neurosphere formation and increased apoptosis. Relevant to this, one more study has shown impaired acquisition and memory following maternal protein restriction during pregnancy and/or lactation. Such memory impairment has been closely associated with altered glucocorticoid production, reduced hippocampal mossy fiber area and decreased basal dendritic length (Reyes-Castro et al., 2018). Additionally, Gianatiempo et al. (2018) have shown that perinatal PMN alters the mother-offspring interaction which further disrupt the maternal behavior and delay the acquisition of developmental landmarks and neurological reflex development. These behavioral alterations were not restricted to F1 generation only but also transmitted to following generation. These recent reports have further strengthened the idea that PMN results in neurobehavioral and epigenetic alteration leading to growth restriction and hypertension (de Brito Alves and Costa-Silva, 2018).

Our group is also engaged in working on the intragenerational PMN model of rats, i.e., on pregestational, gestational, and lactational PMN model (8% protein) which corresponds well with IUGR clinical conditions of poor socioeconomic group of human females. We have reported a compromised physical development (decreased body weight and brain weight), delayed neurological reflex development (cliff avoidance and negative geotaxis reflex), hyperactivity, poor neuromuscular strength, impaired spatial learning and memory and low anxiety in F1 progeny of low protein fed rats (Naik et al., 2015). All these observed behavioral and cognitive impairments are signature mark of neurological disorders including autism and schizophrenia. We have also assessed the astrocytic density and turnover number in LP-F1 progeny using standard immunohistochemical procedures and qRT-PCR assay. Expression of GFAP protein (astrocytic marker) was not evident until E18 in LP rats, whereas numerous stars shaped GFAP+ cells were reported in E18 HP brain which suggested delayed astrogenesis following PMN. The same trend was also recorded in A2B5 (glial restricted precursor) and BLBP (secondary radial glia) immunolabeling where LP brains showed reduced labeling which further indicated low progenitor pooling in the LP-F1 brain (Naik et al., 2017). We further investigated the oligodendrocyte genesis, differentiation, maturation and myelination through immunohistochemistry and quantitative PCR using the expression of myelin associated glycoproteins (MAG), proteolipid protein (PLP), myelin oligo glycoprotein (MOG) and platelet derived growth factor receptor α (PDGFRα) and found reduced expression of myelin proteins depicting impaired myelination and linked behavioral dysfunction following intragenerational protein restricted model (Patro et al., 2018). Our findings clearly demonstrated that the detrimental changes in astrogenesis and oligodendrogenesis were reflected in the neurobehavioral and cognitive outcome in the LP-F1 rats. These results thus support that the early life adversities are the main cause of later life impairments and neurodevelopmental dysfunctions (Anderson et al., 2003; Patro et al., 2013).

## Oxidative Stress in Protein Malnutrition

Oxidative stress generally occurs due to an imbalance between free radical, i.e., pro-oxidant content (hydrogen peroxide, superoxide, hydroxyl radical, alkoxyl and peroxyl radicals) and anti-oxidant (both enzymatic and non-enzymatic) response system of body. This pro-oxidant/anti-oxidant balance is necessary for proper body functioning. Both free radical and mitochondrial theories of aging are the most widely accepted theories of aging which speculate that reactive oxygen species (ROS) alter mitochondrial function by interfering with the replication and transcription machinery of mitochondrial DNA (mtDNA) resulting in more ROS generation, which in turn damage mtDNA. In accordance with the above theories, an aged tissue presents more ROS production suggesting ROS as a critical contributor in aging (Sawada and Carlson, 1987; Sohal and Dubey, 1994; Hamilton et al., 2001; Capel et al., 2005). ROS is responsible for generating DNA lesions which causes genetic instability. The most dominant DNA lesion formed by ROS is 7,8-dihydro-8-oxo-deoxyguanosine (8-oxo-dG) which causes G:C to T:A transversions (Grollman and Moriya, 1993; Dizdaroglu et al., 2002). These devastating outcomes of ROS production can be neutralized by enhancing antioxidant defense system. Numerous studies have shown the inverse relationship between oxidative stress and life span. The enhanced expression of catalase (CAT) and superoxide dismutase (SOD) exhibits extended life expectancy in Drosophila (Orr and Sohal, 1994). Consistently, decreased life span is also observed in those *C. elegans* which are more susceptible to oxidative stress (Larsen, 1993; Ishii et al., 2004) whereas, antioxidant mimetics (SOD/CAT) reverse these changes and enhances *C. elegans* life span (Melov et al., 2000). The increased oxidative stress is also known to promote autophagy in Alzheimer disease (AD), Parkinson disease (PD), amyotrophic lateral sclerosis (ALS), Huntington disease (HD) brain samples, suggesting the possible role of autophagy in the pathophysiology of these diseases (Hirai et al., 2001; Nixon et al., 2005; Rubinsztein et al., 2005; Cherra and Chu, 2008).

Golden and Ramdath (1987), proposed that free radical overproduction is involved in pathogenesis of kwashiorkor leading to haemolytic anemia. Increased oxidative and nitrosative stress is reported to be involved in neurological disorders like AD, PD, HD, and aging (Sies, 1985; Calabrese et al., 2003; Mariani et al., 2005; Valko et al., 2007). de Brito Alves et al. (2016) examined the association between maternal PMN induced hypertension and oxidative stress. They noticed that oxidative dysfunction and impaired antioxidant defense system in ventral medulla might contribute to progression of hypertension following maternal protein restriction. Another study by Verma et al. (2017) also reported increased serum malondialdehyde levels leading to oxidative stress in severe acute malnourished children as compared to control group. Prenatal and lactational PMN in rats is found to be associated with increased levels of thiobarbituric acid reactive substance (lipid peroxidation product) in the cerebellum and cerebral cortex and decreased CAT activity in the cerebellum (Feoli et al., 2006). Similarly, increased plasma malondialdehyde (by-product of lipid peroxidation), protein carbonyl (by-product of protein oxidation) and decreased anti-oxidants (ascorbic acid, glutathione, SOD, ceruloplasmin) were reported in a study conducted over 193 malnourished children of age group 6 months to 5 years in Eastern Uttar Pradesh, India (Khare et al., 2014). Increased red cells SOD was also observed in malnourished children with kwashiorkor and marasmus (Rana et al., 1996; Ashour et al., 1999). However, majority of the studies showed reduced antioxidant activity following PMN (Sive et al., 1993; Houssaini et al., 1997).

In response to any oxidative stress, antioxidant level either decreases due to their depletion during scavenging of free radicals or increases to overcome the oxidative stress development and this shift further depends on multiple factors including type and source of oxidative stress, duration of exposure, toxicant concentration, intensity and model organism. These findings indicate that increased oxidative stress and compromised anti-oxidant defense system following PMN may be a risk factor for developing serious neurological and neurodegenerative disorder at later life.

## Malnutrition and Inflammation

Insufficient dietary intake of nutrients is the leading cause of immunodeficiency worldwide. A synergistic relation exists between malnutrition and infection where nutritional inadequacy increases the risk to infection by impairing both innate and adaptive arms of immune response (Neumann et al., 2004). Infection also affects the nutritional status of an individual by reducing food intake and impairing nutrient absorption (Woodward, 1998; Solomons, 2007). The severity of infection depends on health status, type of infection and dietary intake. PMN is associated with immune impairments that initiate a cascade of inflammatory reaction (Welsh et al., 1998). Undernutrition is also considered as a pro-inflammatory state with increased expression of IL-6 (Dulger et al., 2002). However, PMN induced inflammatory response is still a controversial subject.

Some studies in malnourished children demonstrate a decreased expression of inflammatory markers while others report inflammatory response similar to the healthy children (Bondestam et al., 1988; Malave et al., 1998). Leptin (adipocyte derived cytokine) is considered as a central mediator between nutrition, neuroendocrine system and immunity (Fernandez-Riejos et al., 2010) and PMN has been reported to result in a decreased concentration of leptin, thus increasing the susceptibility to infection (Matarese, 2000). Compromised cellular components of immune response (IFN-Y, TNF-α, nitric oxide) have also been reported following PMN. These components are crucial for protection against *Mycobacterium tuberculosis* (Chan et al., 1996). Breast feeding enhances the infant immunity and provides protection against gastrointestinal and respiratory infections (Chien and Howie, 2001). Epidemiological data favors the association between breast milk feeding and reduced risk of type 1 diabetes, asthma, eczema, rheumatoid arthritis, multiple sclerosis and bowel disease (Brandtzaeg, 2003; Hanson et al., 2003). It contains a variety of lymphocytes, macrophages, neutrophils, cytokines, chemokines, growth factors and long chain polyunsaturated fatty acids (PUFAs) which aid in promoting the neonatal immune system development. Primarily, inflammation is a defensive response against any infection or stress but its exaggerated response may exert negative consequences to the infants. Breast milk is rich in both proinflammatory (IL-1β, IL-6, IL-8, and TNF-α) and anti-inflammatory (IL-10) cytokines. The only limitation in understanding the complete association between breast milk components and infant immunity is the variation that exists amongst women and period of active lactation. Higher fat content is observed in the milk of malnourished women as compared to the well nourished women, although difference is not statistically significant between the two groups (Spring et al., 1985). Alternatively, decreased protein, lactalbumin and lactoferrin concentration is reported in severely malnourished mothers (Lonnerdal et al., 1976; Forsum and Lonnerdal, 1980), whereas high protein diet results in enhanced nitrogen contents of milk (Emery, 1978). The rats fed with a very low protein diet (VLPD) develop systemic inflammation and vascular calcification as revealed in a recent publication (Yamada et al., 2016).

Proinflammatory cytokines in particular TNF-α and IL-6 are reported to affect child growth in chronic inflammatory disorders, which could be rescued when these cytokines are blocked, stressing the correlation of abnormal expression of these cytokines with stunted growth (Sederquist et al., 2014). This was evident from a recently published report by El-Maksoud et al. (2017) demonstrating the increased serum levels of proinflammatory cytokines and C-reactive protein in nutritionally stunted Egyptian children. Thus, there is a possible link between the malnutrition and inflammation, although the exact mechanism by which early life protein deprivation governs neuroimmunity and also how it integrates with neuroendocrine system needs further attention.

## Nutritional Supplementation for Protein Malnutrition

Dietary supplements generally exert protective effects against diet related diseases (obesity, diabetes, cardiovascular disease, and osteoporosis). The successful implementation of nutritional therapies demands a suitable target (pregnant women and/or children), which needs to be properly monitored and supplemented at critical window of development. Diverse studies across the world have proven the positive health benefits of oral nutritional supplementation (ONS) in the subjects suffering from adult malnutrition (Baldwin and Weekes, 2012; Collins et al., 2012). These ONS are enriched in high quality nutrition. Recently it has been reported that ONS improves the muscular strength of leg among malnourished and sarcopenic older patients (Cramer et al., 2016). A number of nutritional interventions during pregnancy were designed and studied to prevent IUGR. Maternal folic acid supplementation either alone or in combination with other vitamins decreases the incidences of neural tube defects in the offspring (Rieder, 1994; Pitkin, 2007; Bhutta et al., 2013). Similarly, maternal intake of cod liver oil during gestational and lactational period is associated with higher intelligence as measured by higher mental processing composite source (Helland et al., 2003). Both oral and intravenous arginine administration in IUGR pregnancies lead to body weight gain in fetus (Sieroszerski et al., 2004; Xiao and Li, 2005). Intra-amniotic or maternal protein supplementation could be a possible way to direct fetal growth. However, only few studies have focused on how maternal intake of individual amino acid or protein affects the embryo-fetal development especially in terms of brain structure, function and behavior.

## Spirulina: A Wonder Nutraceutical

Nowadays, commercial food sector is interested in food or food products with nutritional and medicinal benefits. These types of food products come under an umbrella term ‘nutraceutical.’ They are also called as functional foods, medical foods, nutritional supplements or designer foods. Out of a huge range of available nutrients, microalgae (particularly Chlorella and Spirulina) are gaining special attention as a food supplement due to its easy availability, rapid growth, low cost and high nutritive value. Spirulina is extensively studied for its anti-oxidant, anti-inflammatory, anti-bacterial, anti-viral and immunomodulatory properties (Mallikarjun Gouda et al., 2015; Wu Q. et al., 2016; Finamore et al., 2017) along with its potent role in preventing IUGR related abnormalities as summarized in Table 1.

**Table 1:** Protective effects of Spirulina against oxidative stress and neuroinflammation.

Parameter	Test species	Main findings	Reference
**Protein malnutrition induced oxidative stress and neuroinflammation**			
Oxidative stress	Rats	Increased CAT and decreased SOD activity in marasmic-kwashiorkor rats	Akinola et al., 2010
	Human	Increased lipid peroxidation product (MDA) and decreased anti-oxidant level (GSH, Zn-SOD)	Khare et al., 2014
	Human	Reduced blood levels of CAT, SOD, GSH, vitamin C and increased MDA concentration	Aly et al., 2014


			
	Rats	Increased MDA level, reduced SOD enzyme activity and metabolic dysfunction	Vega et al., 2016
	Human	Increased serum MDA levels	Verma et al., 2017
Neuroinflammation	Mice	Decreased serum protein levels and reduced superoxide anion production	Redmond et al., 1991
	Human	Reduced anti-oxidant level (glutathione and vitamin E) in kwashiorkor patients and increased concentration of IL-6 and soluble receptor of TNF-α	Sauerwein et al., 1997
	Rats	High circulating concentration of TNF-α, increased expression of TNF-α mRNA in liver, reduced phagocytic activity of neutrophils and increased superoxide anion production	Silva et al., 2010
	Rats	Increased serum TNF-α and urinary 8-hydroxydeoxyguanosine level	Yamada et al., 2016
	Human	Increased serum levels of pro-inflammatory cytokines and reduced serum levels of Zn, Ca, and Mg	El-Maksoud et al., 2017

**Anti-oxidant and anti-inflammatory activities of Spirulina**			

Anti-oxidant activity	Human	Reduced plasma level of MDA and increased SOD activity	Lu et al., 2006
	Rat	Increased level of antioxidants (GSH, SOD and CAT)	Jeyaprakash and Chinnaswamy, 2007
	Mice	Decreased lipid peroxidation (LPO) level and antioxidants concentration (SOD and CAT) were restored to near normal level	Sharma et al., 2007
	Mice	Reduced LPO level in hippocampus, striatum and cortex, increased CAT and glutathione peroxidase activity	Hwang et al., 2011
	Rat	Reduced lipid peroxidation and decreased percentage of DNA fragmentation	Hassan et al., 2012
Anti-inflammatory activity	Human	Reduced plasma MDA level, decreased LDL-cholesterol and IL-6 expression	Lee et al., 2008
	Mice	Inhibited humoral and cell mediated immune response and decreased TNF-α production	Rasool and Sabina, 2009
	Human	Increased indoleamine 2,3-dioxygenase (IDO) level and ameliorated senescence	Selmi et al., 2011
	Rat	Reduced expression of TNF-α, IL-1β, and IL-6	Abdel-Daim et al., 2015
	Human	Increased CD4 cell count and significant reduction in viral load	Ngo-Matip et al., 2015


Spirulina is a progeny of first photosynthetic life form that was created by nature 3.6 billion years ago and belongs to the phylum Cyanobacteria. The name Spirulina is derived from a Latin word meaning tiny spiral. It is microscopic, photosynthetic, filamentous, spiral shaped and dark-blue in color due to the presence of pigment called phycocyanin. Surprisingly, it doubles its biomass in every 2–5 days and grows naturally in ponds of brackish or alkaline water. Very few microorganisms are capable of surviving in such extreme conditions in which Spirulina develops which in turn ensures crop hygiene. W.H.O designated it as ‘Food of the Future’ because of its high protein content and rapid growth. Moreover, it is also called as a ‘Super food’ or ‘Wonder food’ and various published scientific studies reveal how it boosts the immune system and improves health. It is approved in Russia as ‘*Medicine food’* for treating radiation induced effects whereas, NASA considered it as a ‘*Best food’* for space travel, as its small quantity contains a range of nutrients.

The most commonly used species of Spirulina are *Spirulina platensis* and *Spirulina maxima.* It has been used as a food for human consumption for centuries and consumed in many different countries such as Germany, Brazil, Spain, France, Canada, United States, Ireland, Philippines, Argentina, India, and Africa. The cell wall of Spirulina is devoid of cellulose and mainly composed of mucopolysaccharides, which makes it easily digested, assimilated and effective for the people suffering from intestinal malabsorption (older people and victims of kwashiorkor). It exhibits various positive biological activities including anti-viral, antibacterial, anti-fungal, anti-parasitic, free radical scavenging (anti-oxidant) and anti-arthritic effect. Our lab demonstrated the protective efficacy of Spirulina against collagen-induced arthritis in (CIA) rats. Various antioxidant constituents (phycocyanin, carotenoids, vitamins) present in Spirulina suppresses the physiological, histological and biochemical changes produced during CIA in rats (Kumar et al., 2009). Use of Spirulina three times a day in fish feed (*Maylandia lombardoi*) is reported to increase growth rate and seed production as compared to Spirulina intake once a day (Karadal et al., 2017). It is widely used in treating nutritional deficiencies, recovering from malnutrition, immune enhancement and in correcting iron anemia. It stimulates hematopoiesis especially erythropoiesis. It meets all international food quality and has applications in health foods and therapeutics. Its impressive protein content and rapid growth have attracted the attention of both researchers and industrialist. Thus, Spirulina supplementation during pregnancy and lactation may be of great potential value as it contains all hematopoietic nutrients that will ultimately benefit both mother and fetus.

## Nutritional Composition of Spirulina

**FIGURE 1:**
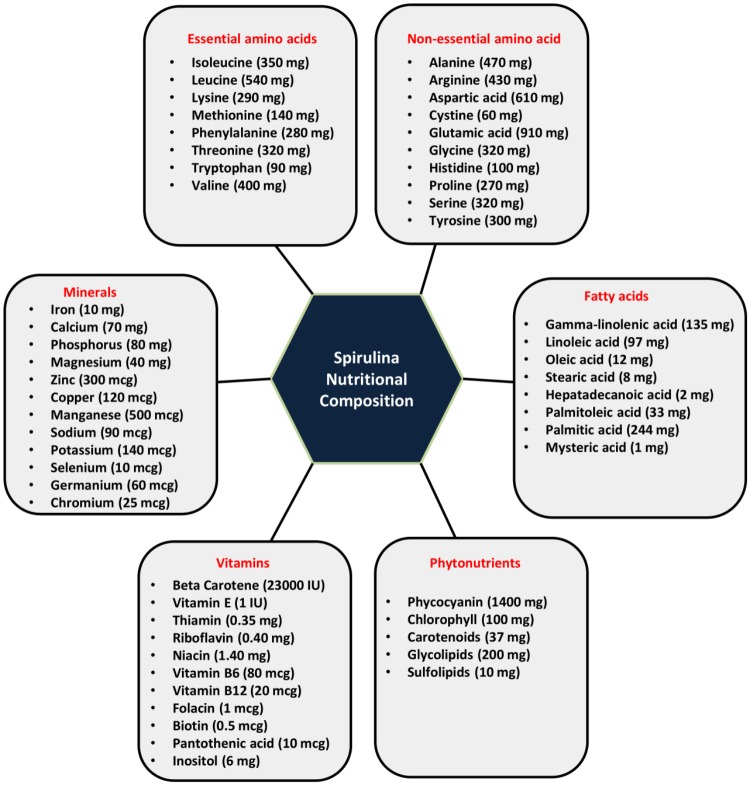
Nutritional composition of Spirulina (serving size-10 gm of Spirulina): Spirulina is incredibly rich in proteins (60–70%) and contains a wide range of essential amino acids, non-essential amino acids, vitamins, minerals, phytonutrients and fatty acids.

Spirulina is one of the most potent sources of nutrition. The protein content of Spirulina varies between 60 and 70% of its dry weight. It also contains vitamins (vitamin B-12, beta carotene, vitamin E), various mineral substances (iron, calcium, phosphorus, magnesium, and trace minerals), essential fatty acids (gamma-linoleic acid, palmitic acid, linoleic acid, oleic acid, etc.), polysaccharides (rhamnose and glycogen), glycolipids and sulfolipids, enzymes (SOD responsible for quenching free radicals) and various pigments like phycocyanin, chlorophyll, carotenoids (Campanella et al., 1999; Blinkova et al., 2001; Watanabe et al., 2002; Colla et al., 2004; Khan et al., 2005; Earthrise, 2006) as represented in Figure 1. Phycocyanobilin (phycobilin-protein complex) is an inhibitor of NADPH oxidase. This enzyme is involved in oxidative stress in various neurological disorders. Thus, Spirulina intake decreases the activity of NADPH oxidase and has therapeutic interventions in many vascular diseases, cancers, diabetes, neurodegenerative and inflammatory disorders (McCarty, 2007). It has been shown that carbohydrates present in Spirulina increases cell nucleus enzyme activity (particularly endonucleases) and DNA repair synthesis (Baojiang, 1994). It also positively influences both the humoral (antibodies and cytokines) and cell-mediated immunity (T cell and macrophages). Downregulation of inflammatory and oxidative stress markers is observed in rats with Spirulina rich diets both in aging and neurodegenerative disorders making it more suitable as a natural drug for the treatment of neurological disorders.

## Spirulina as Therapeutic Intervention Against Inflammation and Oxidative Stress in Aging and Neurodegenerative Conditions

The predominant factors responsible for aging and neurodegeneration are inflammation and oxidative stress. There is a decline in the normal antioxidant and anti-inflammatory defense mechanisms in both aging and neurodegeneration that makes the brain more susceptible to the deleterious effects of oxidative stress (Finkel and Holbrook, 2000). There are considerably strong evidences elucidating that most neurological disorders (AD, PD, HD, ALS, inflammatory injuries, and senility) are the result of oxidation and/or inflammation. Various nutraceuticals as well as pharmaceuticals have been extensively investigated for their anti-inflammatory and anti-oxidant potential. With respect to nutraceutical, several dietary supplementations (blueberries, spinach and Spirulina) have been reported to protect CNS by downregulating the markers of inflammation and oxidative stress and thus reducing neurological deficits. Spirulina consumption also improves life span with numerous health benefits, increases locomotor activity and reduces HSP70 (indicator of cellular stress) and Jun-N-terminal kinase signaling (JNK signaling involved in modulating the life span) in DJ-1β^Δ93^ flies (a Parkinson’s disease model) in *Drosophila melanogaster* (Kumar et al., 2017). A myriad of studies demonstrate that this dietary supplementation increases cerebellar glutathione levels, reduces malondialdehyde levels, decreases pro-inflammatory cytokines and improves spatial and motor learning in senile rats (Bickford et al., 2000; Gemma et al., 2002).

Thus, although an immense literature is available specifying the widely documented use of Spirulina as a functional food, yielding health promoting properties and/or reducing the risk of disease (Wu Z. et al., 2016; Furmaniak et al., 2017) only a very few and discrete studies have focused to report the adverse effects of Spirulina. More recently, the genome and proteome analysis of *Arthrospira platensis* has clearly mentioned the absence of genes responsible for the synthesis of various toxins (Furmaniak et al., 2017), supporting the statement that Spirulina shows no toxicity, i.e., neither acute nor chronic, making it safe for the human use (Gutierrez-Salmean et al., 2015). However, the minor adverse effects of Spirulina, reported include headache, gastrointestinal discomforts, muscle pain and cramps, skin rashes, etc. (Iwasa et al., 2002; Mazokopakis et al., 2008; Marles et al., 2011). In one study, the Spirulina was reported to be a causative factor for acute rhabdomyolysis in a young human patient (Mazokopakis et al., 2008). Although we have not come across any more studies specifying the adverse effects of Spirulina, but keeping in view the increasing use of Spirulina as dietary supplement, more studies are warranted to focus this issue.

## Spirulina as a Neuroprotectant

Phenotypic outcomes are generally governed by epigenetic processes suggesting a possible connection between food quality and neurological disorders. Neuroprotective effects of Spirulina are well evidenced in ischemic brain damage with progressive decline in TUNEL positive cells and caspase-3 activity in the ischemic hemisphere (Wang et al., 2005). Brain ischemia or cerebral ischemia is a condition marked by the cerebral hypoxia that leads to the generation of free radicals, reactive oxygen or nitrogen species and energy crisis. Phycocyanin and phycocyanobilin present in the Spirulina have strong anti-cyclooxygenase-2 and anti-oxidant activities that reduce peroxynitrite induced oxidative damage to DNA (Bhat and Madyastha, 2001). Further advances and intervention studies in omics technology may provide useful information in understanding the mechanism of microglia mediated neuroinflammation (Patro et al., 2016) and the possible role of nutritional approaches in regulating microglia aging (Wu Z. et al., 2016).

Dietary supplementation with Spirulina in rat model of Parkinson’s disease results in significant reduction in lesion volume and decreased microglial activation (Stromberg et al., 2005). Anti-inflammatory effects of Spirulina have also been investigated against LPS-induced inflammation in rodent model. LPS insult causes increased astrogliosis with prominent activation of GFAP in existing cells and decreased proliferation of neural progenitor cells (NPCs). However, diet supplemented with 0.1% Spirulina for 28 days before LPS administration prevents the LPS induced decrease in NPC proliferation and astrogliosis (Bachstetter et al., 2010). Researchers at University of Yaounda (Azabji-Kenfack et al., 2011) found that food supplementation with Spirulina for 12 weeks in malnourished adults infected with HIV stimulates weight gain and increase fat free mass as compared to soya beans. In addition, anti-retroviral treatment (ART) along with Spirulina showed more beneficial effects than ART coupled to soyabeans (increased CD4 cell counts and decreased viral load in Spirulina group). Spirulina platensis was also found to suppress the peripheral sensitization, improve motor coordination and restore motor activity in collagen-induced arthritic rats by reducing NF-200 accumulation in spinal cord neurons suggesting a possible neuroprotective role of Spirulina for the treatment of rheumatoid arthritis (Patro et al., 2011). Spirulina also supports the viability of astrocytes (Kim et al., 2012). Interestingly, polycaprolactone Spirulina nanofiber mat (composite nanomedicine) was proved to be effective against CNS injury as it reduces the astrocyte activation which in turn, could reduce inflammation induced by astrogliosis. Neuroprotective role of Spirulina is also marked in alpha-synuclein model of Parkinson’s disease, where increased expression of tyrosine hydroxylase (TH) positive and NeuN positive cells was observed. Accordingly, reduced number of activated microglia was also reported as determined by the reduced OX6 (MHC-II) immunostaining (Pabon et al., 2012).

**FIGURE 2:**
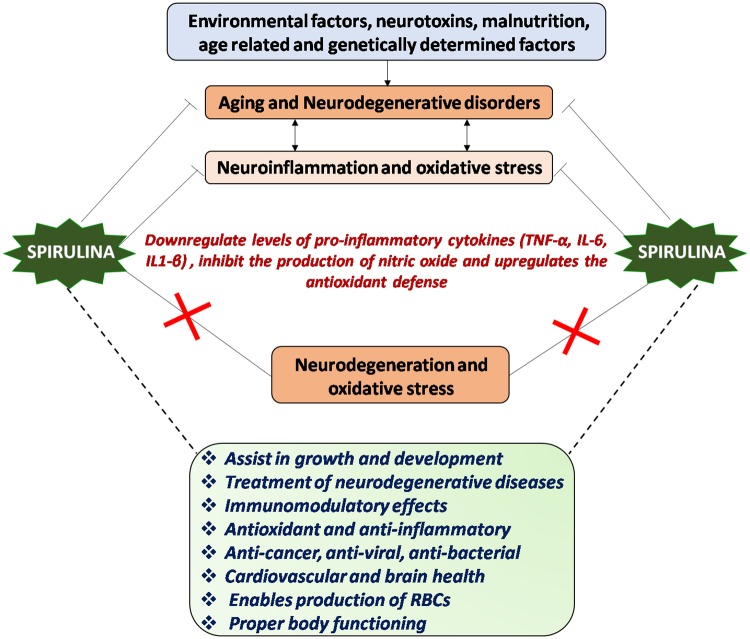
Mechanism of action of Spirulina: Environmental and genetic factors regulate aging process and progression of neurological disorders, chiefly characterized by neuroinflammation and oxidative stress or vice-versa. Spirulina has both antioxidant and anti-inflammatory activities and downregulates the proinflammatory cytokines, which in turn might inhibit the neurodegeneration and oxidative stress thereby aids in maintaining proper brain and body health.

It is thus apparent that dietary complementation with Spirulina could be beneficial to the patients suffering from neurodegenerative disorders. It maintains proliferation, differentiation and migration of NPCs which may lead to improved brain functioning and body health. The elevated response of human stem cells in terms of proliferation potential was also reported in Spirulina fed group. In normal conditions, CX3CL1 and CX3CR1 are expressed at high levels in brain, but as age advances their expression decreases. Spirulina intake shows the increased expression of CX3CR1, suggesting a possible mechanism of action in neuroprotection (Pabon, 2011). Generally, non-steroidal anti-inflammatory drugs act by suppressing the immune activation but Arthrospira enhances both the innate and adaptive immunity thereby increasing cellular and humoral adaptive immunity. There are now accumulating evidence that constituents of Spirulina have both anti-oxidant and anti-inflammatory activities that inhibits ROS formation and decreases the cytokine mediated neuroinflammation, thereby making it a suitable and effective therapeutic target for combating neurodegenerative disorders (Figure 2).

## Nutritional Rehabilitation Using Spirulina – Super Food to Fight Against Malnutrition

Hunger can be either due to the non-availability of food or due to the lack of micronutrients. Inadequate food sources and improper nutritional awareness program in developing countries are the major causes of malnutrition. High protein content of Spirulina makes it a suitable adjunct to combat malnutrition. Both *Spirulina platensis* and *Spirulina maxima* have been extensively studied due to its high protein content, micronutrient composition, vitamins and minerals, easy and rapid reproducibility, inexpensive and non-toxic nature. Mass culture and utilization of Spirulina as a potent protein source against hunger, malnutrition and starvation was first explored and published by Rodulfo (1990). Many studies have shown a strong association between malnutrition and anemia thus suggesting malnutrition as a common cause of anemia in both children and elderly (Mitrache et al., 2001; Anticona and San Sebastian, 2014; Sahin et al., 2016).

Numerous studies have been conducted to test the efficacy of Spirulina supplementation in rodent model covering a range of variables including body growth, protein efficiency ratio (PER), hematological status and toxicity (Maranesi et al., 1984; Tranquille et al., 1994; Salazar et al., 1996; Narayan et al., 2005). Enhanced skeletal muscle proteins were also reported in Spirulina supplemented young rats (Voltarelli and de Mello, 2008). These animal based studies were further extended to clinical studies in human subject. In this context, various non-government organizations (NGO) and international organizations have chosen to work for reducing public health problems and prevention of malnutrition in developing countries. Azabji Kenfack and associates in 2011 examined the effect of daily Spirulina intake for 12 weeks on 56 malnourished HIV infected adult patients and concluded that Spirulina consumption effectively improved weight and body mass index (BMI) among undernourished HIV sufferers. Spirulina supplementation at a dose of 10 gm per day was found to improve the nutritional status as well as to increase the corpuscular hemoglobin and hematocrit levels in malnourished children in Democratic Republic of the Congo (Matondo et al., 2016). Another research on 550 malnourished children showed the beneficial effects of Spirulina in combination with Misola (millet, coja, and peanut). This combination was proved to be more effective than Spirulina or Misola alone (Simpore et al., 2006). In addition to these, Spirulina platensis also exerts a positive impact against immunosenescence and anemia because of the presence of active components such as folic acids, vitamin B12, phycocyanin, essential amino acids and iron content, which in turn play central role in Erythropoiesis (Kapoor and Mehta, 1998; Mani et al., 2000; Khan et al., 2005; Selmi et al., 2011). Subsequently, one more study was conducted in Burkina Faso evaluating the effects of Spirulina consumption on 84 HIV positive and 86 HIV negative anemic children, and the results indicated that 81.8% of HIV negative and 63.6% HIV positive children recovered from anemia, thus speculating potent role of Spirulina even in patients with compromised immune system (Simpore et al., 2005). Effectiveness of Spirulina against child malnutrition has also been reported in a study in Zambia, where 10 gm daily Spirulina consumption significantly improved HAZ (height-for-age-z) score in malnourished children (Masuda et al., 2014). From the above reports, it can be safely inferred that Spirulina supplementation can effectively combat the malnutrition.

## Neuroprotective Effects of Spirulina Consumption During Pregnancy And/Or Lactation

Majority of the available studies have demonstrated the neuroprotective effects of Spirulina in adult animals. However, only few studies have been designed to investigate how maternal Spirulina supplementation would influence the developmental profile of an offspring. Spirulina enriched diet (0.1% Spirulina) to lactating mothers, 1 day prior to LPS treatment in offspring maintains the p38 and IL-1β levels thereby regulating neuroinflammation and antioxidant defense system in the offspring (Patil et al., 2018). Furthermore, Spirulina consumption by pregnant women in Dakar region is proved to be more effective than iron and folic acid (IFAC) supplementation in terms of weight gain and improved hemoglobinemia in the newborn (Niang et al., 2016). It has also been reported that supplementing pregnant hyperglycemic albino mice with Spirulina improves fertility rate, reproductive performance and reduces teratogenicity associated with diabetes (Pankaj, 2015). Furthermore, Banji et al. (2013) have reported that consumption of this edible alga from embryonic day (ED) 6 to postnatal day (PND) 15 reduces fluoride toxicity in developing brain, promotes antioxidant formation and minimizes the risk of neurodevelopmental disorders. These evidences mark the beneficial health effects of Spirulina in offsprings following Spirulina supplementation to pregnant mothers. However, the exact mechanism by which Spirulina supplementation during pregnancy imparts health benefits to the offsprings remains to be elucidated.

To the best of our knowledge, there is no complete study evaluating how Spirulina supplementation to malnourished pregnant and lactating mother would affect the oxidative functioning, inflammatory response and mental skills among children. Thus, enriching maternal environment with potent protein source (Spirulina) can be an effective way to reduce oxidative stress, neuroinflammation, behavioral and cognitive deficits induced by maternal PMN. This strategy of using Spirulina during pregnancy and lactation period would be advantageous for treating abnormalities during IUGR pregnancy.

## Conclusion and Recommendation

Any environmental stress during critical periods of CNS development affects the developmental profile of an individual. Brain homeostasis and neuronal communication may be disturbed specifically in conditions of protein deprivation. Increased reactive oxygen and nitrogen species, impaired antioxidant defense system, altered glial cell physiology and inflammatory response are postulated to be the possible drivers in PMN induced neurocognitive decline. Diets enriched in foods with high ORAC could be used in reverting age and poor diet associated behavioral, cognitive and neurochemical impairments and maintaining cellular homeostasis. Spirulina contains a combination of nutrients (β carotene, vitamin B12, tocopherols, essential fatty acids, polysaccharides, glycolipids, sulfolipids and phycobiliprotein) which exerts more neuroprotective effects than single nutrient source. The present review has compiled the numerous studies conducted on Spirulina to establish its implications as a potent source of nutrition to combat against micronutrient deficiency, PMN and neurological disorders. The data discussed in this review suggests that exposure to malnutrition during critical developmental timeline where developmental plasticity is at peak, results in long lasting irreversible behavioral and cognitive abnormalities which further increases the risk of neurological disorders.

**FIGURE 3:**
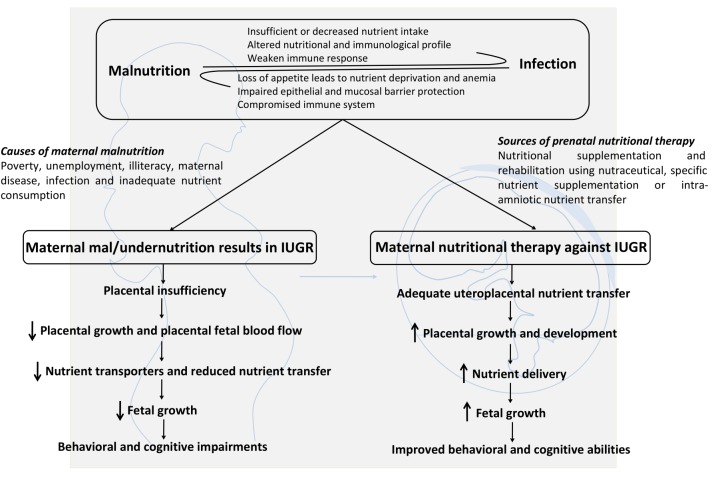
Maternal malnutrition causes, consequences and possible nutritional supplementation strategies to treat IUGR: A synergistic relationship exists between malnutrition and infection. Maternal malnutrition results in decreased body growth and impaired behavioral and cognitive abilities. Prenatal nutritional therapy represents a promising approach to treat IUGR by enhancing uteroplacental nutrient transfer, placental growth and nutrient transport, fetal growth which ultimately results in improved behavioral and cognitive abilities.

However, this review has certain limitations. More research is needed in understanding how Spirulina consumption affects neuron-glia communication? What is the exact molecular mechanism of Spirulina action? Is Spirulina supplementation enough for completely combating the detrimental effects of malnutrition? What are the important subcomponents of Spirulina necessary for making it as a neuroprotective agent and its dose response? What are the possible molecular targets while considering pharmaco-therapeutic applications of Spirulina? Does it exert any effect on human epigenome? How Spirulina supplementation regulates placental function? All these questions need to be resolved in order to make Spirulina as an ideal natural drug with neuroprotective properties. Various nutritional inputs including nutritional supplementation, rehabilitation and therapy, nutritional education and specific nutrient supplementation (vitamins, mineral, and micronutrient) are necessary to overcome the detrimental effects of IUGR for better fetal growth and development (Figure 3). A combination of nutrition education and nutritional supplementation could exert more beneficial effects than any of the mentioned nutritional inputs alone. Inconsistency in research studies focusing on prenatal therapies and long term nutritional intervention programs are the major barriers to design effective strategies to fight malnutrition. Thus, a special attention should be given to animal studies involving both pre- and early postnatal nutritional supplementation along with intra-amniotic nutrient transfer strategies to ameliorate fetal growth and metabolic functioning during IUGR pregnancy. Further advances and elucidation of the mechanism of action of dietary supplements and their effects on microbiota-gut brain axis will therefore open up new windows for therapeutic intervention against neurological disorders arising from malnutrition.

## Author Contributions

SS wrote this review with input from NP and IP.

## Conflict of Interest Statement

The authors declare that the research was conducted in the absence of any commercial or financial relationships that could be construed as a potential conflict of interest.
